# One Health Investigation of SARS-CoV-2 Infection and Seropositivity among Pets in Households with Confirmed Human COVID-19 Cases—Utah and Wisconsin, 2020

**DOI:** 10.3390/v13091813

**Published:** 2021-09-12

**Authors:** Grace W. Goryoka, Caitlin M. Cossaboom, Radhika Gharpure, Patrick Dawson, Cassandra Tansey, John Rossow, Victoria Mrotz, Jane Rooney, Mia Torchetti, Christina M. Loiacono, Mary L. Killian, Melinda Jenkins-Moore, Ailam Lim, Keith Poulsen, Dan Christensen, Emma Sweet, Dallin Peterson, Anna L. Sangster, Erin L. Young, Kelly F. Oakeson, Dean Taylor, Amanda Price, Tair Kiphibane, Rachel Klos, Darlene Konkle, Sanjib Bhattacharyya, Trivikram Dasu, Victoria T. Chu, Nathaniel M. Lewis, Krista Queen, Jing Zhang, Anna Uehara, Elizabeth A. Dietrich, Suxiang Tong, Hannah L. Kirking, Jeffrey B. Doty, Laura S. Murrell, Jessica R. Spengler, Anne Straily, Ryan Wallace, Casey Barton Behravesh

**Affiliations:** 1 Centers for Disease Control and Prevention, 1600 Clifton Road, Atlanta, GA 30329, USA; nrm9@cdc.gov (C.M.C.); krr4@cdc.gov (R.G.); wpb7@cdc.gov (P.D.); kuo2@cdc.gov (C.T.); mwi4@cdc.gov (J.R.); oew5@cdc.gov (V.M.); pgz4@cdc.gov (V.T.C.); pha6@cdc.gov (N.M.L.); wyz0@cdc.gov (K.Q.); fwz9@cdc.gov (J.Z.); pln9@cdc.gov (A.U.); wul2@cdc.gov (E.A.D.); sot1@cdc.gov (S.T.); hrj7@cdc.gov (H.L.K.); uwb7@cdc.gov (J.B.D.); wjl9@cdc.gov (L.S.M.); wsk7@cdc.gov (J.R.S.); yzv2@cdc.gov (A.S.); euk5@cdc.gov (R.W.); dlx9@cdc.gov (C.B.B.); 2 Animal and Plant Health Inspection Service, Veterinary Services, United States Department of Agriculture, 2150 Centre Avenue, Bldg B., Fort Collins, CO 80526, USA; jane.a.rooney@usda.gov; 3 National Veterinary Services Laboratories, Animal and Plant Health Inspection Service, Veterinary Services, United States Department of Agriculture, 1920 Dayton Ave, Ames, IA 50010, USA; mia.kim.torchetti@usda.gov (M.T.); christina.m.loiacono@usda.gov (C.M.L.); mary.l.killian@usda.gov (M.L.K.); melinda.jenkins-moore@usda.gov (M.J.-M.); 4 Wisconsin Veterinary Diagnostic Laboratory, University of Wisconsin-Madison, 445 Easterday Ln, Madison, WI 53706, USA; Ailam.Lim@WVDL.wisc.edu (A.L.); keith.poulsen@wvdl.wisc.edu (K.P.); dan.christensen@wvdl.wisc.edu (D.C.); emma.sweet@WVDL.wisc.edu (E.S.); 5 Utah Department of Health, 288 N 1460 W, Salt Lake City, UT 84116, USA; dietdr.pete@gmail.com (D.P.); annasangster@utah.gov (A.L.S.); eriny@utah.gov (E.L.Y.); koakeson@utah.gov (K.F.O.); 6 Utah Department of Agriculture and Food, 350 N Redwood Rd, Salt Lake City, UT 84116, USA; djtaylor@utah.gov (D.T.); amandaprice@utah.gov (A.P.); 7 Salt Lake County Health Department, 788 Woodoak Ln, Murray, UT 84107, USA; mkiphibane@slco.org; 8 Wisconsin Department of Health Services, 1 W Wilson St, Madison, WI 53703, USA; Rachel.Klos@dhs.wisconsin.gov; 9 Wisconsin Department of Agriculture, Trade and Consumer Protection, 2811 Agriculture Dr, Madison, WI 53718, USA; Darlene.Konkle@wisconsin.gov; 10 City of Milwaukee Health Department Laboratory, 841 N Broadway, Milwaukee, WI 53202, USA; sbhatt@milwaukee.gov (S.B.); dtrivikram@hotmail.com (T.D.)

**Keywords:** COVID-19, SARS-CoV-2, pets, dog, cat, transmission, zoonoses, household transmission

## Abstract

Approximately 67% of U.S. households have pets. Limited data are available on SARS-CoV-2 in pets. We assessed SARS-CoV-2 infection in pets during a COVID-19 household transmission investigation. Pets from households with ≥1 person with laboratory-confirmed COVID-19 were eligible for inclusion from April–May 2020. We enrolled 37 dogs and 19 cats from 34 households. All oropharyngeal, nasal, and rectal swabs tested negative by rRT-PCR; one dog’s fur swabs (2%) tested positive by rRT-PCR at the first sampling. Among 47 pets with serological results, eight (17%) pets (four dogs, four cats) from 6/30 (20%) households had detectable SARS-CoV-2 neutralizing antibodies. In households with a seropositive pet, the proportion of people with laboratory-confirmed COVID-19 was greater (median 79%; range: 40–100%) compared to households with no seropositive pet (median 37%; range: 13–100%) (*p* = 0.01). Thirty-three pets with serologic results had frequent daily contact (≥1 h) with the index patient before the person’s COVID-19 diagnosis. Of these 33 pets, 14 (42%) had decreased contact with the index patient after diagnosis and none were seropositive; of the 19 (58%) pets with continued contact, four (21%) were seropositive. Seropositive pets likely acquired infection after contact with people with COVID-19. People with COVID-19 should restrict contact with pets and other animals.

## 1. Introduction

SARS-CoV-2, the cause of the coronavirus disease 2019 (COVID-19) pandemic, likely originated in bats [1]. Threats from pathogens shared by humans and animals highlight the need for a One Health approach for detection, prevention, and control [2]. One Health is a collaborative, multisectoral, and transdisciplinary approach with the goal of achieving optimal health outcomes recognizing the interconnection between people, animals, plants, and their shared environment. 

In the United States (U.S.), approximately 85 million households (67%) own ≥1 pet, with dogs (63 million households) and cats (43 million households) being most popular [3]. Human-animal interactions are associated with improved mental, social, and physiologic health [4] and are critical for people with service and working animals [5]. 

Some animals, including pets, have been naturally infected with SARS-CoV-2, almost exclusively after exposure to an infected person [6,7,8]. Dogs, cats, ferrets, hamsters, and rabbits are pet species with demonstrated susceptibility to SARS-CoV-2 infection under experimental conditions. Cats, ferrets, and hamsters can transmit the virus to naïve cohabitants of the same species [9,10,11,12,13,14]. SARS-CoV-2 has also been reported as a contaminant on pet fur [15,16] and animal health and welfare concerns have been reported [17,18], including reports of misuse of cleaning products on pets to the Pet Poison Hotline (R. Schmid, personal communication). 

We conducted a One Health household transmission investigation to better characterize SARS-CoV-2 infection in mammalian pets living in households with people with COVID-19 to inform guidance and decision-making during this pandemic and for future preparedness efforts.

## 2. Materials and Methods

### 2.1. Participant Enrollment

The U.S. Centers for Disease Control and Prevention (CDC) collaborated with local and state public health and agriculture departments in Utah and Wisconsin, Wisconsin Veterinary Diagnostic Laboratory (WVDL), and USDA to conduct a One Health investigation that enrolled mammalian pets from an ongoing COVID-19 household transmission investigation that included households with ≥1 person with laboratory-confirmed COVID-19 captured by public health surveillance, previously described [17]. The investigation enrolled human index COVID-19 patients (hereafter addressed as index patients) and household contacts in March 2020 from 62 households to determine secondary household infection rates over a 14-day follow up period since household enrollment. Convenience sampling was used to select households for this investigation. Eligible households required that the index patient: (1) was not hospitalized at the time of enrollment, (2) lived with ≥1 additional person, and (3) tested positive for SARS-CoV-2 by real-time reverse-transcription polymerase chain reaction (rRT-PCR) from a nasopharyngeal swab collected ≤10 days prior to enrollment. All persons within the household were asked to participate and households where >1 person declined were excluded [17]. Detailed epidemiologic, clinical, and exposure information was collected for all human household members; most human household members had respiratory specimens collected for SARS-CoV-2 viral testing and blood for serology testing at ≥2 time points. Physical characteristics of each residence, including size, were also described [17]. Human household members with nasopharyngeal or nasal swabs positive by rRT-PCR or who seroconverted during the investigation were classified as lab-confirmed SARS-CoV-2 infection [17]; additionally, human household members reporting any symptoms since illness onset of the index case were considered symptomatic.

Of 62 enrolled households, 41 households with ≥1 mammalian pet living in the household were eligible for inclusion in this One Health investigation (Figure S1). Eligible households were contacted by phone during March–April 2020. Pets were enrolled if owners consented, a questionnaire was completed, and ≥1 sample was collected from each pet. Phone interviews were conducted prior to initial home visits to identify pet species residing in the home and whether the pet(s) developed clinical signs consistent with SARS-CoV-2 infection after the index patient’s COVID-19 diagnosis. 

### 2.2. Household Visits

Initial household visits for pet sampling occurred between April–May 2020 after enrollment in this investigation. Pet sampling was conducted in coordination with repeat visits for the human investigation where possible. During the first household visit for pet sampling, CDC field teams administered a questionnaire (Appendix A) to capture information on each pet’s demographics, past medical history, household knowledge of public health recommendations, and the following variables for the pet after the index patient’s illness onset: clinical signs; household and community interactions; and household and personal precautionary measures taken. Frequent daily contact was defined as having a duration of interaction >1 h/day between the index patient and the pet (range: 1–>12 h). Households were also given an educational information sheet on animals and SARS-CoV-2 (Appendix B).

During household visits, veterinarians collected oropharyngeal, nasal, rectal, and fur swabs, feces, and blood from pets. Bilateral deep nasal, oropharyngeal, and rectal swabs were collected using sterile polyester tipped swabs (tip diameter, 1.981 mm for nasal, 5.2 mm for oral and rectal). Swabs were placed into 3 mL of brain heart infusion broth. Fur swabs were collected in duplicate using 2 × 2-inch sterile gauze pads rubbed across the back and the abdomen, as well as the dorsal and ventral paws and between the metacarpal and digital pads of each pet. One sample was stored dry and one was stored in RNAlater (Thermo Fisher Scientific, Waltham, MA, USA). All samples, except for dry fur swabs and fecal samples, were stored on ice packs for immediate shipping and were processed for testing upon arrival at WVDL (Madison, WI, USA). Dry fur swabs and fecal samples were placed in containers without media and were frozen immediately at −80 °C until testing. Serum samples were obtained from venous blood (1–3 mL) collected and processed in serum separator tubes; sera were decanted and stored at −80 °C until testing. 

### 2.3. rRT-PCR and Serology of Animal Specimens

Preliminary RNA extraction and rRT-PCR testing of animal specimens occurred at WVDL (Appendix C). If rRT-PCR was positive at WVDL for either target, the sample was considered a presumptive positive and sent to the national animal reference laboratory, USDA’s National Veterinary Services Laboratories (NVSL; Ames, IA, USA) for confirmatory testing per the USDA Case Definition (https://www.aphis.usda.gov/animal_health/one_health/downloads/SARS-CoV-2-case-definition.pdf accessed on 13 May 2020). One dry fur swab, the duplicate of the positive fur swab stored in RNAlater, was forwarded to NVSL for confirmatory testing, including rRT-PCR, sequencing, and viral culture attempts (Appendix C). The positive fur swab stored in RNAlater was forwarded to CDC to attempt sequencing (Appendix C). Serum neutralizing antibodies were assessed at NVSL by a SARS-CoV-2 virus neutralization (VN) assay (Appendix C). Neutralizing titers of 1:8–1:16 were considered suspect in the absence of other positive findings; titers >1:16 were considered seropositive. 

### 2.4. Analysis 

Characteristics of enrolled pets, risk factors for seropositivity, number of human cases and household infection rates, and clinical features of human cases within households were analyzed using SAS version 9.4 (SAS Institute, Cary, NC, USA). Clopper-Pearson (exact) method was used to calculate 95% confidence intervals for seropositivity rates. Features of households with and without seropositive pets were compared using Mann-Whitney-Wilcoxon tests.

## 3. Results

Initial household visits for pet sampling occurred from 0–32 days (median: 14 days) after enrollment in the household transmission investigation. Fifty-six pets (37 dogs, 19 cats) from 34 of 41 eligible (83%) households were enrolled (Figure S1; Table 1); 21 households had only dog(s), seven households had only cat(s), and six households had dogs and cats. Median household size was four people (range: 2–8) and one pet (range: 1–5) (Table 2). The median proportion of human household members with laboratory-confirmed COVID-19 was 45% (range: 13%–100%); of 72 total people with confirmed infection, 71 (99%) ever experienced symptoms. Additional household characteristics are described in Table 2. 

Fifty-six pets (100%) had oral and fur swabs, 55 (98%) had nasal swabs, 54 (96%) had rectal swabs, 14 (25%) provided fecal samples, and 47 (84%) provided blood samples. Fourteen pets had repeat oral, nasal, rectal, and fur swabs, six had repeat fecal samples, and 11 had repeat blood samples. 

The median time from symptom onset of the index patient to first date of pet sampling was 27 days (range: 3–46 days; Table 2). The median time from first positive diagnostic result of the index patient to first date of pet sampling was 20.5 days (range: 3–42 days) and was similar between households with and without seropositive pets (21.5 vs. 20 days). 

**Figure 1:**
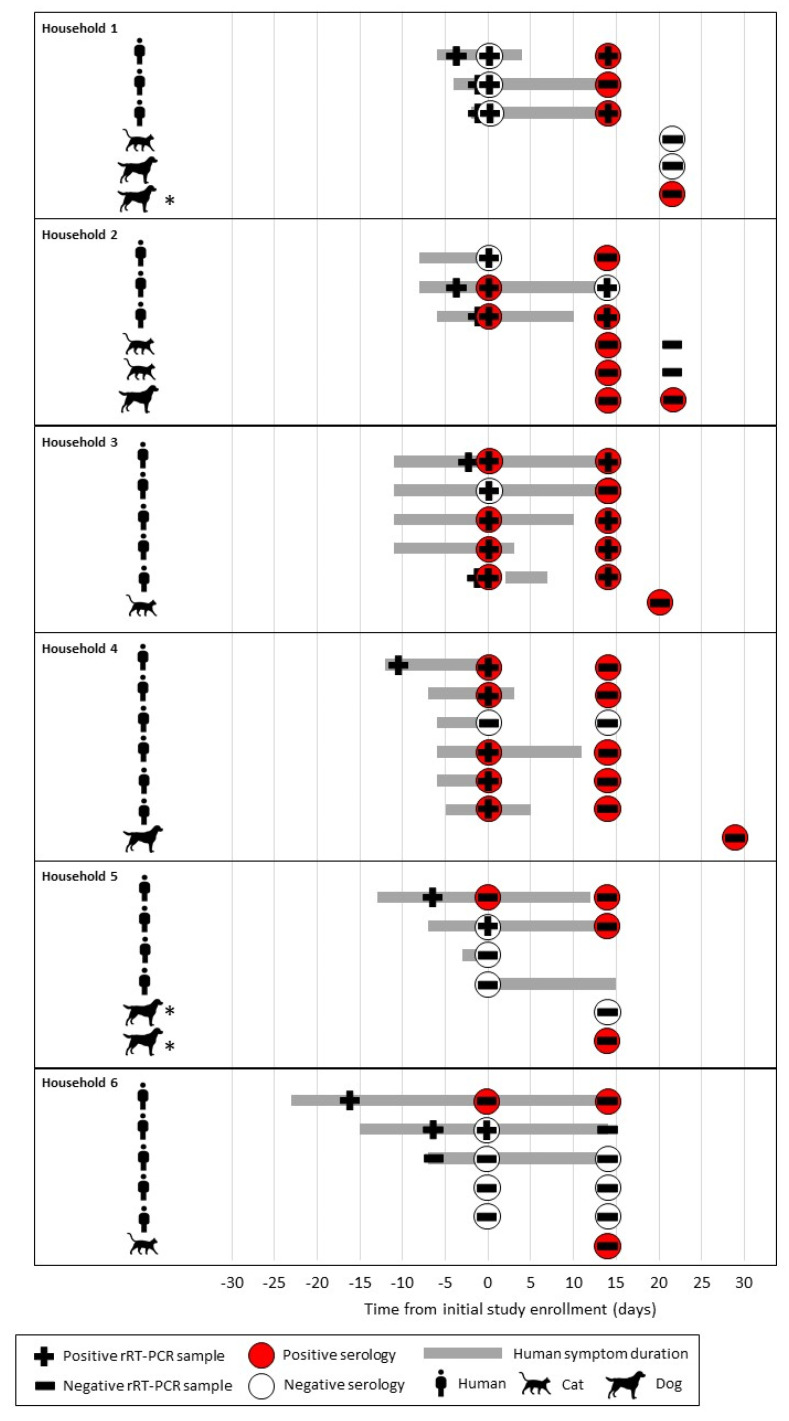
COVID-19 diagnostic testing and symptom duration among humans and animals in households with a seropositive pet, One Health COVID-19 Household Transmission Investigation, April–May 2020. Symptom durations are shown only for humans. Pets with clinical signs are denoted with an *.

All oropharyngeal, nasal, and rectal swabs and fecal specimens tested negative by rRT-PCR, except one rectal swab sample from a cat was presumptive positive that was not confirmed (Appendix D; Table S1). Among 47 pets with serological results from 30 households, eight pets (17%; four dogs, four cats) from six (20%) households, had detectable SARS-CoV-2 neutralizing antibodies. Three pets from these six households had seronegative results. The neutralizing titers for all seropositive dog samples were 32 while cat titers ranged from 32 to 128 (Table S1). Demographic pet data by serology result are presented in Table 1. Timelines for human and animal sample collection among households with seropositive pets, as well as symptom onset and duration in people in those households, are depicted in Figure 1. 

**Figure 2:**
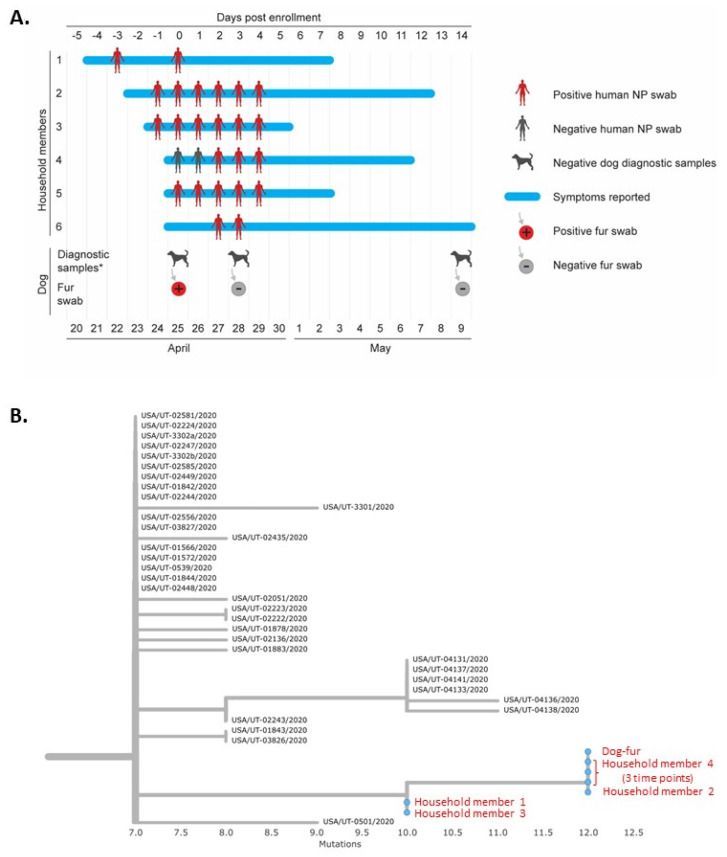
Timeline and phylogenetic analysis of human and dog testing in one household in Utah with SARS-CoV-2 RNA detected on the dog’s fur, One Health COVID-19 Household Transmission Investigation, April–May 2020. (**A**) Timeline of human and dog testing in one household in Utah with six persons with laboratory-confirmed COVID-19 and SARS-CoV-2 RNA detected on the dog’s fur. The timeline indicates dates of reported symptoms and results of nasopharyngeal swab testing by rRT-PCR in human COVID-19 cases and samples collected from the dog in the household. Diagnostic samples * from the dog included oral, nasal, and rectal swabs and stool, which all tested negative by rRT-PCR, and a blood sample which was negative by virus neutralization. (**B**) Enhanced view of branch-tip from comprehensive phylogram (Figure S2), depicting here the seven study sequences (red) alongside selected Utah complete genome sequences available from Global Initiative on Sharing All Influenza Data. Branch length is by divergence. See Figure S2 for zoomed-out dendrogram depicting additional available sequences from Utah.

SARS-CoV-2 RNA was detected from duplicate fur swabs from one of 56 pets (2%) at the first pet sampling visit and subsequent fur swabs from this dog were negative (Figure 2). The day the positive fur swab was collected, all six human household members reported symptoms consistent with COVID-19. Five people had nasopharyngeal swabs collected on that day, and four were positive by rRT-PCR. The person who was initially not tested and the one who was initially negative were tested two days later, both were positive (Figure 2). 

Seven near-complete or complete-genomes were generated from this household; one each from humans 1–3, three from human 4 collected at three time-points, and one consensus sequence from the dog fur swabs. High sequence similarity suggests one introduction from the community and subsequent internal household transmission (Figure 2 and Figure S2). Notably, the dog had no evidence of infection; all samples were negative by rRT-PCR and the dog was also seronegative (Figure 2). Viral culture was attempted on the rRT-PCR positive fur swab but was negative.

Owners reported clinical signs consistent with SARS-CoV-2 infection among 14 (25%) pets during the time from symptom onset of the index patient until time of sampling (Table S2). The most reported clinical signs were respiratory (16%), including sneezing (7%), coughing (7%) and nasal discharge (5%). Among the eight seropositive pets, clinical signs were reported in only two (25%); one dog had nasal discharge and one dog had decreased appetite. Among 39 seronegative pets, clinical signs were reported in eight (21%) (Table S2).

**Figure 3:**
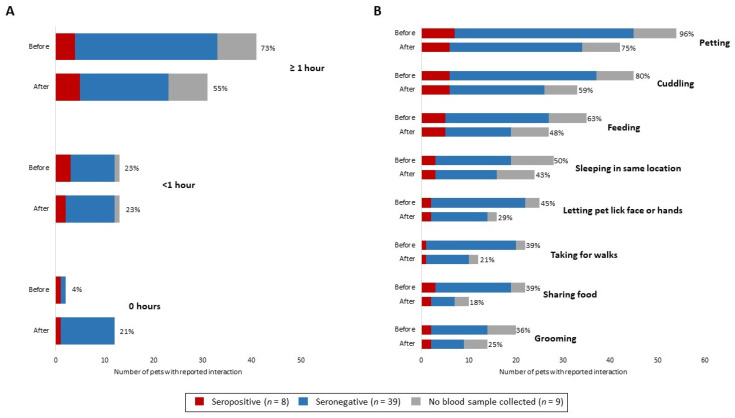
(**A**) Reported duration of interaction per day and (**B**) types of interactions between human index patients and pets in each household before and after human index patient diagnosis, by pet serostatus—One Health COVID-19 Household Transmission Investigation, April–May 2020.

Forty-six (98%) of 47 pets with serological results were primarily indoor pets; one pet, an 8-year-old seropositive cat, spent ≥50% time outdoors (Table 1). Seropositivity among pets occurred more commonly among households with higher rates of secondary transmission among people; the median proportion of people with laboratory-confirmed COVID-19 in households with a seropositive pet was 79% (range: 40–100%) compared to 37% in households with no seropositive pet (range: 13–100%) (*p* = 0.01) (Table 2). Overall, owners reported pets had fewer daily interactions lasting ≥1 h and fewer types of interaction with the index patient after their COVID-19 diagnosis; interactions included petting, cuddling, feeding, sleeping in the same location, pets licking the index patient’s face or hands, taking for walks, sharing food, and grooming (Figure 3). 

Among the 47 pets with serologic results, 33 (70%) pets were reported to have frequent daily contact (≥1 h) with the index patient before the person’s diagnosis. Of 14 pets with decreased interactions, none (0%) were seropositive. Nineteen pets continued to have frequent contact with the index patient after their diagnosis; of these, four (21%) were seropositive. 

Five (15%) of 34 households, comprising 12 (21%) pets, reported that, after their COVID-19 diagnosis, the index patient began wearing face masks and two (6%) also reported glove use around pets. In households using face masks, among pets with serological results, one of eight (13%) pets was seropositive, while in households not using face masks, seven of 39 (18%) pets were seropositive.

Of 34 households, 10 (29%) identified a household member familiar with CDC recommendations for people with suspected or confirmed COVID-19 restricting contact with pets [18]; three (30%) of the 10 households had a seropositive pet. Of the 10 households familiar with CDC recommendations, implementation of precautions was low; the index patient in one (10%) household reduced interactions with pets after the person’s diagnosis, one (10%) household used masks and gloves while interacting with pets, and one (10%) household reported both reduced interaction and mask and glove use. 

## 4. Discussion

The epidemiologic role of pets in the COVID-19 pandemic is not fully understood. This One Health investigation systematically evaluated pets residing in households with people with laboratory-confirmed COVID-19. At the time this investigation began, three countries had reported natural SARS-CoV-2 infection in 11 animals, including household pets [8,19,20]. This investigation identified a similar rate of SARS-CoV-2 seropositivity (17%) among pets living in households with human COVID-19 cases to a subsequent study in the United States (19%) [16]; these studies in the United States both identified higher rates of seropositivity compared to previously published studies from Italy (10%) and France (0%) [21,22]. The 12% seropositivity rate in dogs with serological results in our investigation was similar to other studies [16,21]; however, the 31% seropositivity in cats is higher than most studies [7,16,21,23]. 

While 25% of pets were reported to have clinical signs consistent with SARS-CoV-2 infection, no animals received veterinary treatment specific to these signs. Only two seropositive animals identified were reported to have mild clinical signs consistent with SARS-CoV-2 infection during the period when the infection was most likely. Similar clinical signs were reported in the seronegative pets at a similar frequency. Clinical signs consistent with SARS-CoV-2 infection in animals are generally non-specific and could potentially be attributed to other factors. Cross-species zoonotic transmission events are documented, but are likely under-recognized because of asymptomatic pet infections, small sample sizes, and few published studies with variable results [21,22]. 

In our investigation, more seropositive pets were found in households with a greater rate of human household secondary transmission. Further investigations are needed to evaluate SARS-CoV-2 transmission dynamics between people and pets including anthropogenic or mechanical factors such as whether isolation precautions were taken or infectious dose was altered by differences in viral shedding; architectural differences among homes; ventilation system usage patterns affecting air flow; environmental cleaning; or personal protective equipment use. Analysis of household prevention measures, such as facemask use by index patients, was limited by small sample sizes in this study; further investigations are needed to characterize the effectiveness of these measures to prevent SARS-CoV-2 transmission to pets.

Several seropositive animals identified roamed freely in the yard or neighborhood during their likely infectious window, which raises questions on the potential for transmission of virus from infected pets to people and susceptible animals, which is biologically plausible, but has not yet been documented. One seropositive pet cat was reported to have spent ≥50% of its time outdoors. While several studies have reported that seropositivity of SARS-CoV-2 among stray cats was not found or was very low [24,25,26], experimental studies have documented that cats with SARS-CoV-2 can transmit SARS-CoV-2 to other cats [13,27], leading to concerns of transmission between cats that roam outdoors; however, this was not assessed in this investigation. 

We detected SARS-CoV-2 RNA in fur swabs collected from only one dog but were not able to culture the virus from these samples. Thirty (54%) pets were sampled at a time when at least one household member was symptomatic and 14 (25%) pets at a time when at least one household member tested positive; therefore, some environmental contamination from human viral shedding may have been missed. Our findings suggest that viral RNA on the fur was due to environmental contamination from human household members. Our investigation, along with other studies [16,28], further highlight that there is no evidence that pet fur can serve as a fomite for SARS-CoV-2 transmission. 

In households where the index patient decreased duration of interaction with pets after the person’s diagnosis, no pets in this study were seropositive. In two households with seropositive pets, the index patient increased their duration of interaction with pets after their diagnosis (Figure 3). This finding highlights the importance of people with suspected or confirmed COVID-19 restricting contact with pets and other animals to prevent person-to-animal transmission, in accordance with CDC recommendations [29]. 

We identified 10 households with awareness of CDC’s recommendations of restricting interactions with pets for people with COVID-19 [29] before enrollment. While this metric was captured only at a single time point, it emphasizes the importance of providing accurate and timely health protection messaging for pets during a pandemic caused by an emerging zoonotic disease. 

Our findings provide additional characterization of potential SARS-CoV-2 transmission from people with laboratory-confirmed COVID-19 to pets in households; however, several limitations are noted. While directionality cannot be proven based on these results, the epidemiological information gathered, in conjunction with what is currently known about disease course and shedding of SARS-CoV-2 in companion animals, suggests that human infection preceded animal infection. In experimental infection studies, viral RNA was detected up to the study endpoint—12 days post-infection for cats [9,12,13], while only on day 6 for dogs [9]. However in cases of natural infection, viral RNA was detected up to 14 and 19 days in dogs [6] and cats [30,31], respectively, post confirmatory testing of the index patient. In this investigation, the median time from symptom onset of the index patient to specimen collection was 27 days (range: 3–46 days) and the median time from first positive diagnostic result of the index patient to specimen collection was 20.5 days (range: 3–42 days), which would have missed the shedding window for infected pets and could explain the lack of viral RNA detection. The time to pet sampling from the index patient’s symptom onset and from diagnosis were similar among households with and without seropositive pets, and therefore, most pets had a similar length of time to mount neutralizing antibody responses since the beginning of their exposure to the household’s human case(s). Additionally, the sample size of enrolled and tested pets was insufficient to allow for definitive conclusions regarding risk factors for pet infection and to compare interactions between pets and index patients.

Future investigations of household transmission should aim to sample pets across the spectrum of exposure, including time points closer to the start of the index patient’s exposure window, if possible, and at multiple subsequent time points to learn more about viral shedding, symptomatology, and risk factors. Further One Health efforts are needed to better understand the risk of SARS-CoV-2 transmission between people and pets and to further characterize the course of SARS-CoV-2 infection in pets, both of which will inform guidance and decision-making to best protect public health, animal health, and welfare. 

This investigation shows that transmission of SARS-CoV-2 from people to pets can occur in household settings. We identified a similar rate of seropositivity of SARS-CoV-2 among pets as another subsequent study in the United States. Given the relative frequency of human-to-animal transmission in households with people with COVID-19, people with confirmed or suspected COVID-19 should restrict contact with pets and other animals [18]. If a person must care for their pet while they are sick, they should wear a mask and should wash their hands before and after interacting with them [18]. 

## 5. Conclusions

A One Health approach for the prevention and control of SARS-CoV-2 [2,32], as well as other emerging and zoonotic diseases, is critical, including response and surveillance efforts to capture and assess transmission dynamics between people, animals, and their shared environment. Previous zoonotic and infectious disease investigations have highlighted the importance of including pets in household transmission investigations. This One Health investigation provides additional evidence that pets can be infected with SARS-CoV-2, especially after contact with people with COVID-19. People with confirmed or suspected COVID-19 should restrict contact with pets and other animals. Further One Health efforts are needed to characterize SARS-CoV-2 infection in pets and further understand the epidemiologic role pets have in the pandemic, however based on information available to date, the risk of pets spreading COVID-19 to people appears low. Pets contribute to people’s health and well-being, and proper prevention measures to limit microbial transmission between people and pets should be taken to prevent zoonotic infections.

## Figures and Tables

**Table 1:** Characteristics of household pets enrolled in the One Health COVID-19 Household Transmission Investigation, April–May 2020.

Characteristics	Total N (Column %)	Blood Sample Collected		No Blood Sample Collected *n* (Row %)
		Seropositive ^1^ *n* (Row %)	Seronegative *n* (Row %)	
Total	56	8 (14)	39 (70)	9 (16)
Study site				
Utah	38 (68)	6 (16)	31 (82)	1 (3)
Wisconsin	18 (32)	2 (11)	8 (44)	8 (44)
Species				
Dog	37 (66)	4 (11)	30 (81)	3 (8)
Cat	19 (34)	4 (21)	9 (47)	6 (32)
Age (years)				
<2	11 (20)	1 (9)	7 (64)	3 (27)
2–9	33 (59)	5 (15)	23 (70)	5 (15)
≥10	12 (21)	2 (17)	9 (75)	1 (8)
Sex and reproductive status				
Male	29 (52)	4 (14)	19 (66)	6 (21)
Neutered	23 (79)	3 (13)	16 (70)	4 (17)
Female	27 (48)	4 (15)	20 (74)	3 (11)
Spayed	22 (81)	4 (18)	15 (68)	3 (14)
Indoor/outdoor housing environment				
Primarily indoors	55 (98)	7 (13)	39 (71)	9 (16)
Primarily outdoors ^2^	1 (2)	1 (100)	0	0
Exposures outside of the household setting ^3^				
Spent any time free-roaming in the yard or the neighborhood	29 (52)	5 (17)	21 (72)	3 (10)
Attended a social setting (e.g., dog park, daytime boarding facility, veterinary clinic)	5 (9)	0	5 (100)	0

^1^ Serologic testing was conducted using a SARS-CoV-2 virus neutralization assay. Neutralizing titers greater than 16 were considered seropositive. ^2^Defined as spending >50% time outdoors. ^3^ Includes exposures documented after the household human index patient began isolation.

**Table 2:** Characteristics of humans with SARS-CoV-2 infection, household members, and timing of human illness in households with pets enrolled in the One Health COVID-19 Household Transmission Investigation, April–May 2020.

Characteristics	Total Households N = 34	Households with ≥1 Seropositive ^1^ Pet *n* = 6	Households with Seronegative Pet(s) Only *n* = 24	Households with No Pet Blood Sample Collected *n*= 4	*p*-Value ^2^
**Human SARS-CoV-2 infection and timing**					
	**Median (range)**				
Proportion of human household members ^3^ with laboratory evidence of SARS-CoV-2 infection ^4^	0.45 (0.13–1.00)	0.79 (0.40–1.00)	0.37 (0.13–1.00)	0.63 (0.25–1.00)	0.01
Days from symptom onset in the human index patient to first date of pet sampling	27 (3–46)	28 (22–39)	24 (3–46)	32.5 (24–42)	0.30
Days from first positive diagnostic result of the human index patient to first date of pet sampling	20.5 (3–42)	21.5 (18–38)	20 (3–41)	25.5 (21–42)	0.37
**Household members and size**					
	**Median (range)**				
No. persons ^5^	4 (2–8)	4·5 (3–6)	4 (2–8)	3 (2–4)	0.70
No. dogs and cats ^6^	1 (1–5)	1·5 (1–3)	1 (1–5)	1 (1–2)	0.47
Total square meters	213.68 (55.74–706.06)	181.16 (90.95–315.87)	241.55 (55.74–706.06)	192.40 (130.06–260.13)	0.24

^1^ Serologic testing was conducted using a SARS-CoV-2 virus neutralization assay. Neutralizing titers greater than 16 were considered seropositive. ^2^ Households with and without a seropositive pet by Mann-Whitney-Wilcoxon test. ^3^ Includes only household members enrolled in the COVID-19 Household Transmission Study; some household members declined participation. ^4^ Includes individuals positive on nasopharyngeal or nasal swabs by rRT-PCR or with SARS-CoV-2 antibodies detected. ^5^ Includes all persons residing in the households, regardless of study enrollment. ^6^ Pets of other species were not assessed in this analysis.

## Data Availability

Complete or near-complete genome sequences of SARS-CoV-2 obtained in this investigation are available at Global Initiative on Sharing All Influenza Data (GISAID) and GenBank. Additional information or de-identified data may be made available to researchers who submit a methodologically sound proposal to the corresponding author.

## Supplementary Materials

The following are available online at https://www.mdpi.com/article/10.3390/v13091813/s1, Figure S1: Enrollment and sampling of household pets in the One Health COVID-19 Household Transmission Investigation, April–May 20, Figure S2: Phylogenetic tree with selected Utah complete genome sequences available (as of 15 July 2020) from Global Initiative on Sharing All Influenza Data, Table S1: Information on seropositive pets and their households from the One Health COVID-19 Household Transmission Investigation, April–May 2020, Table S2: Owner-reported clinical signs among household pets enrolled in the COVID-19 Household Transmission Investigation since onset of illness in first household human case, April–May 2020.

## Appendix A

Animal questionnaire used during the One Health COVID-19 Household Transmission Investigation, April–May 2020.

## Appendix B

Information sheet on animals and SARS-CoV-2 provided to households with pets enrolled in the One Health COVID-19 Household Transmission Investigation, April–May 2020.

## Appendix C

Supplementary Methods.

### Appendix C.1. RNA Extraction and rRT-PCR of Animal Specimens

Preliminary RNA extraction and real-time reverse-transcription polymerase chain reaction (rRT-PCR) testing of animal specimens occurred at WVDL. To evaluate fecal samples, a swab of feces was obtained and suspended in 1 mL PBS. For all swab specimens, RNA was extracted from 50 µL of sample (in brain heart infusion media or PBS) along with Xeno RNA (10,000 copies, Thermo Fisher Scientific) using the MagMAX-96 Viral RNA Isolation Kit (Thermo Fisher Scientific) on a 96-well KingFisher Flex extraction platform and eluted in a volume of 50 µL according to manufacturer’s instructions. 5 µL of extracted RNA was used for a one-step rRT-PCR targeting the 2019-nCoV N gene sequences (N1 and N2). The rRT-PCR assay was based on CDC assay [33] with alternative reagents using the 2019-nCoV RUO Kit (Catalog # 10006713 Integrated DNA Technologies, Coralville, IA, USA), the AgPath-ID One-Step rRT-PCR Reagents and the VetMAX Xeno Internal Positive Control- LIZ assay (Thermo Fisher Scientific). The rRT-PCR amplification was performed with 1 cycle at 50 °C for 15 min and 90 °C for 10 min, followed by 40 cycles of 95 °C for 15 s and 55 °C for 1 min on a 7500 Fast Real-Time PCR Instrument (Applied Biosystems, Carlsbad, CA, USA).

At NVSL RNA was extracted from 50 µL of sample using the MagMAX-96 Viral RNA Isolation Kit (Thermo Fisher Scientific) on a 24-well KingFisher extraction platform and eluted in a volume of 90 µL according to manufacturer’s instructions. A modified CDC one-step rRT-PCR N-target assay [N1 and N2 targets] [19,33] was used on an Applied Biosystems 7500 Fast Real-Time PCR Instrument according to Emergency Use Authorization instructions for use. Specimens testing presumptive positive at WVDL and confirmed by NVSL were considered SARS-CoV-2–positive. An inconclusive result refers to when only one of two targets (N1 or N2 gene sequences) of the rRT-PCR assay was detected [33].

### Appendix C.2. Virus Neutralization

For VN, 25 µL of two-fold serially diluted sera (for final dilutions of 1:8 to 1:512) were pre-incubated with 25 µL of 100 TCID50/mL of SARS-CoV-2 (2019-nCoV/USA-WA1/2020) in MEM-E containing 200 UI/mL penicillin, 200 µg/mL streptomycin, 75 µg/mL gentamicin sulfate and 6 µg/mL Amphotericin B for 60 min at 37 °C with 5% CO_2_. Each serum sample was tested in duplicate in 96-well plates. At one-hour post-infection, 150 µL of Vero 76 cells were added to the virus-serum mixtures. The neutralization titers were determined at three days post infection. The titer was recorded as the reciprocal of the highest serum dilution that provided 100% neutralization of the reference virus, as determined by visualization of cytopathic effect. Of approximately 620 sera from cats and dogs, 27% have tested positive by the VN at NVSL. The specificity of the VN assay was assessed in-house by testing sera with antibodies to transmissible gastroenteritis, porcine epidemic diarrhea virus, porcine hemagglutinating encephalomyelitis virus, bovine coronavirus, and Aleutian disease. Additional specificity testing was conducted on a known panel of 47 sera with antibodies to common feline and canine coronaviruses. All of this testing was negative by the VN assay.

### Appendix C.3. Genome Sequencing and Analysis

At CDC, nucleic acid from four rRT-PCR positive specimens (dog fur stored in RNAlater and three specimens from household member 4 from various time points) was extracted and sequenced using the MinION (Oxford Nanopore Technologies, Oxford Science Park, England) and MiSeq (Illumina, San Diego, CA, USA) following previously published protocols [4] and consensus sequences were generated with Minimap 2.17 and Samtools 1.9. Missing gaps after MinION and MiSeq sequencing were filled by individual rRT-PCR followed by Sanger sequencing. Consensus complete genome sequences were generated using Sequencher 5.4.6.

At the Utah Public Health Laboratory (Salt Lake City, UT, USA), residual nucleic acid extractions from rRT-PCR positive specimens from household members 1, 2, and 3 were used for input for the ARTIC amplicon sequencing protocol (Josh Quick 2020. nCoV-2019 sequencing protocol. www.protocols.io/view/ncov-2019-sequencing-protocol-v2-bdp7i5rn (accessed on 7 June 2020)). The amplicons generated were then sequenced on the Illumina MiSeq platform. Consensus sequences were generated using UPHL’s custom analysis workflow Cecret (https://github.com/UPHL-BioNGS/Cecret accessed on 23 July 2020).

RNA sequencing on the dry dog fur swab was performed at NVSL using the Ion AmpliSeq Kit for Chef DL8 and Ion AmpliSeq SARS-CoV-2 Research Panel (Thermo Scientific) and sequenced using an Ion 520 chip on the Ion S5 system using the Ion 510™ & Ion 520™ & Ion 530™ Kit. FASTQ files were shared with CDC to include in analysis.

Representative complete genome sequences were downloaded on 15 July 2020 from GISAID, and phylogenetic relations were inferred using maximum likelihood analyses implemented in TreeTime using the Nextstrain pipeline.

### Appendix C.4. Viral Culture of Fur Swab

Viral culture was performed in Vero cells (ATCC CCL-81) under biosafety level-3 conditions. Cells were cultured in minimum essential medium with Earle’s balanced salt solution (MEM-E) with 5% fetal bovine serum (FBS), 25 µg/mL gentamicin sulfate, and 2 µg/mL amphotericin B (growth media). Cells were seeded in T25 flasks for at least 48 h.

Specimens were diluted 1:2 in MEM-E containing 200 UI/mL penicillin, 200 µg/mL streptomycin, 75 µg/mL gentamicin sulfate, and 6 µg/mL amphotericin B. Cells were inoculated with approximately 1.5 mL of the diluted sample and adsorbed for 1 h at 37 °C. Mock-inoculated cells were used as negative controls. After adsorption, cells were washed three times with MEM-E, replacement medium was added, cells were incubated at 37 °C and monitored for cytopathic effect (CPE) once daily for up to seven days. Cell cultures with no CPE were frozen, thawed, and subjected to up to two blind passages, with inoculation of fresh cultures with the lysates as described above. Virus isolation was confirmed in the cell cultures by SARS-CoV-2-specific rRT-PCR using the CDC N1 and N2 primer and probe sets [33].

## Appendix D

Supplementary Results.

### Appendix D.1. Unique Medical Histories and Clinical Signs

One pet was reported to have an immunocompromising condition; this was a 14-year-old cat with feline immunodeficiency virus (FIV). This pet was negative for SARS-CoV-2 on serologic testing. Two pets were reported to receive immunosuppressive medications (corticosteroids and Janus kinase inhibitor, respectively) at the time of sampling; both pets were negative on serologic testing.

### Appendix D.2. Sequence Analysis of Household UT-36 Human Samples and Dog Fur Swab

Seven near-complete genomes or complete-genomes were generated from household UT-36; three from household member 4, collected at three different time points, three from household members 1–3, and one from the dog’s fur swab. High quality sequences were not recovered from household members 5 and 6, and were excluded from analysis. All sequences from household UT-36 formed a unique closely related or identical cluster that nested within many sequences from the United States, including sequences in the major clade containing the S: D614G mutation [34], characteristic of many sequences from United States and Europe (Figure S2). Household member 4 was sampled across three time points, yielding identical sequences. Household member 1, the index patient, had an identical sequence to member 3, and was separated from sequences from members 2 and 4 by two mutations (Figure 1). A nearly complete genome was also generated from a dog fur swab, which was identifical to members 2 and 4 (Figure 1). High similarity in sequences suggests a single introduction from the community and internal transmission within the same household, UT-36.

### Appendix D.3. Presumptive Positive Sample

One rectal swab from one cat collected 18 days after the first positive test of a human case in the household was presumptive positive (Ct of 36) at WVDL. During confirmatory testing at NVSL, only one of the two targets that define a positive result was detected (SARS-CoV-2 N2 target; Ct 39.6) and sequence could not be attempted. Virus isolation was negative for this rectal swab. SARS-CoV-2 neutralizing antibodies were detected in this cat’s blood sample (titer of 128) (Table S1).
