# Supplementary material for: One Health Investigation of SARS-CoV-2 Infection and Seropositivity among Pets in Households with Confirmed Human COVID-19 Cases—Utah and Wisconsin, 2020
Source: Viruses. 2021 Sep 12;13(9):1813. doi: 10.3390/v13091813 (PMC8472995; doi:10.3390/v13091813)
Supplement: Supplementary file 1 [file viruses-13-01813-s001.zip › viruses-1343089-supplementary.pdf]

Supplementary Files

One Health Investigation of SARS-CoV-2 Infection and Seropositivity among Pets in Households with Confirmed Human COVID-19 Cases — Utah and Wisconsin, 2020

Figure S1. Enrollment and sampling of household pets in the One Health COVID-19 Household Transmission Investigation, April–May 2020

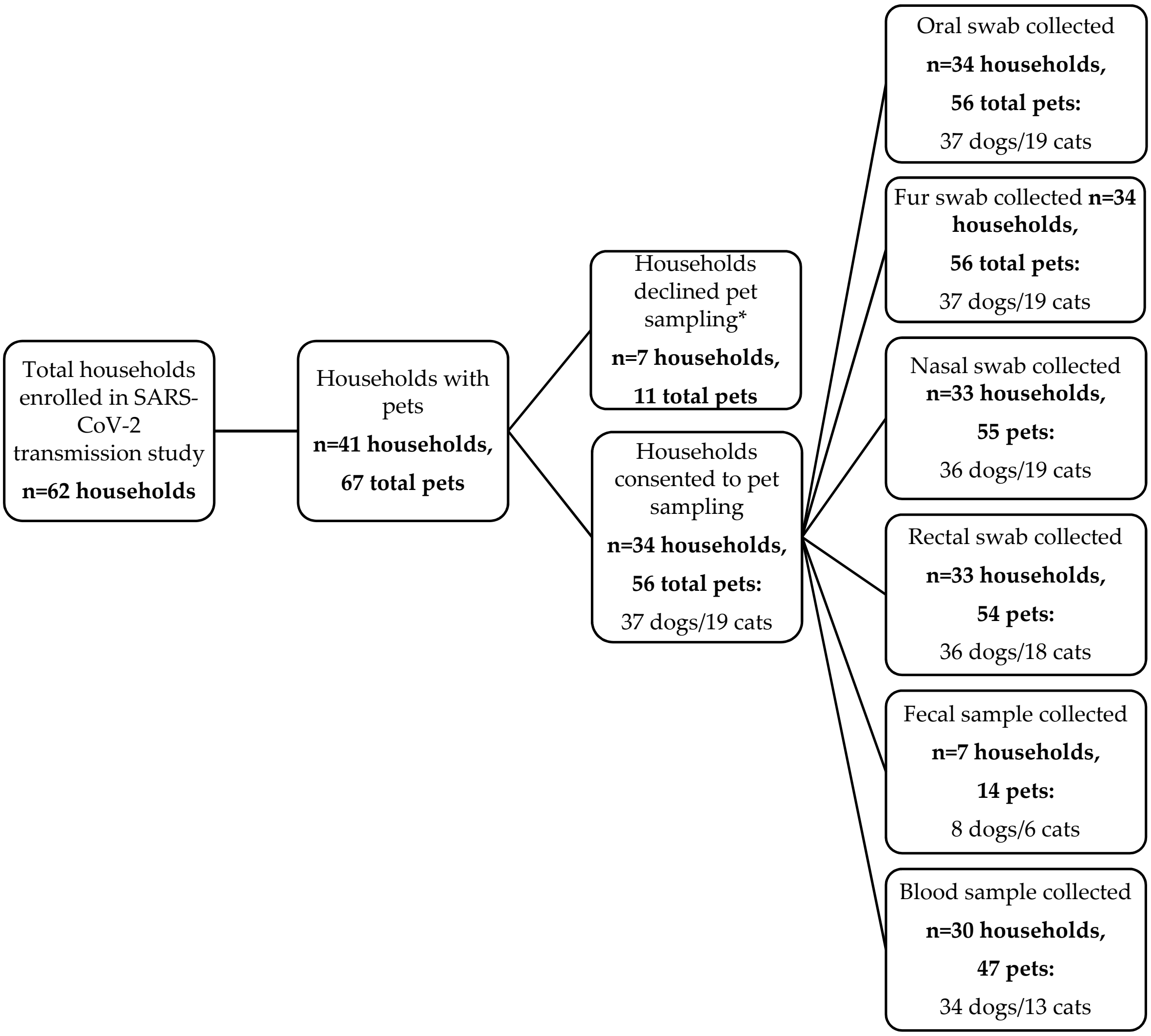

*Households that declined pet sampling expressed safety concerns for the veterinary field teams due to their pet being fractious.

Figure S2. Phylogenetic tree with selected Utah complete genome sequences available (as of July 15, 2020) from Global Initiative on Sharing All Influenza Data, color-coded by subclade (19A – 20C); the seven study sequences are shown in red. All study sequences fell within the 20A subclade with the S: D614G mutation. Branch length denotes time interval.

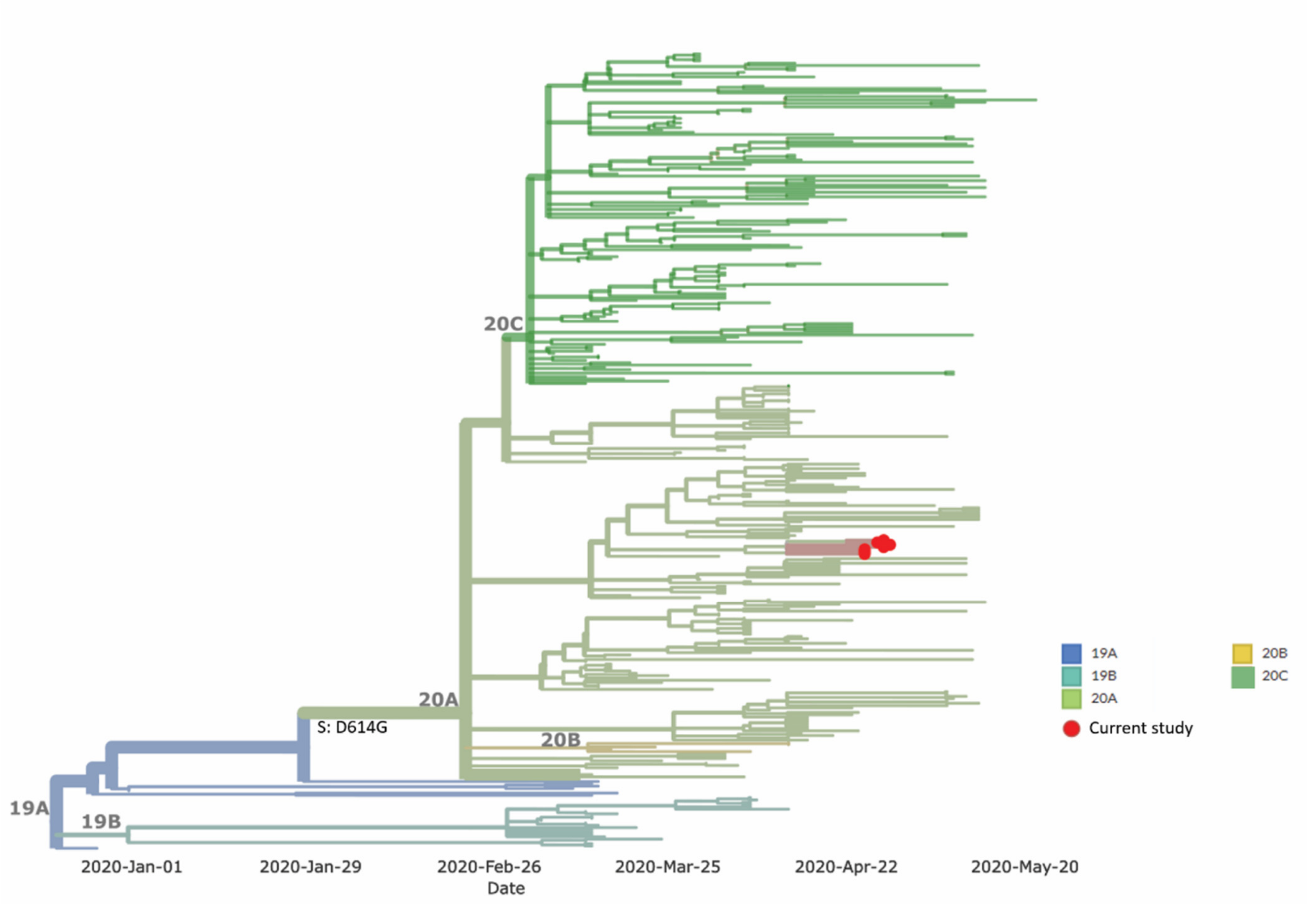

Table S1: Information on seropositive pets and their households from the One Health COVID-19 Household Transmission Investigation, April–May 2020

Household	Pet	Species	Sex [*]	Age (years)	# days sample(s) was collected after symptom onset of the household index patient	# days sample(s) was collected after household enrollment	Serostatus (days post-enrollment)	Pre-Existing Condition	Clinical signs[†]	Notes
1	Dog	Australian Shepherd/ Labrador Retriever mix	MC	2	27	21	Seropositive (1:32)	-	Decreased appetite	All three human household members had at least one positive rRT-PCR result on nasopharyngeal swabs and were seropositive.
	Dog	German Shepherd	FS	12	27	21	Seronegative	-	-	
	Cat	Domestic shorthair	FS	14	27	21	Seronegative	-	-	
2	Cat	Domestic longhair	MC	6	22, 29[+], 33	14, 21[+], 25	Seropositive (1:64) on day 14 and 25	-	-	All three human household members had at least one positive rRT-PCR result on nasopharyngeal swabs and were seropositive. One rectal swab from the 6-year-old female cat was presumptive positive but was not confirmed at NVSL and was negative by virus isolation.
	Cat	Domestic medium-hair	FS	6	22, 29[+], 33	14, 21[+], 25	Seropositive (1:128) on day 14 and 25	Prediabetic; history of asthma	-	
	Dog	Miniature Schnauzer	FS	12	22 and 29	14 and 21	Seropositive (1:32) on day 14 and 21	-	-	
3	Cat	Domestic shorthair	MC	8	31	20	Seropositive (1:64)	-	-	All five human household members had at least one positive rRT-PCR result on nasopharyngeal swabs and were seropositive.
4	Dog	Golden Retriever/Poodle mix	MC	10	39	29	Seropositive (1:32)	15-year history of seizures	-	Four of five human household members had at least one positive rRT-PCR result on nasopharyngeal or nasal swabs and were seropositive; one was seronegative without a positive swab result.
5	Dog	Pitbull	FS	3	27	14	Seropositive (1:32)	-	Nasal discharge	Two of four human household members had at least one positive rRT-PCR result on nasopharyngeal or nasal swabs and were seropositive; two were negative on initial swabs and serology, and convalescent specimens were not obtained.
	Dog	Pitbull	MC	1	27	14	Seronegative	-	Nasal discharge	
6	Cat	Manx	FS	8	37	14	Seropositive (1:64)			This cat spent at least 50% time outdoors. Only one of five human household members was seropositive; an additional member had positive rRT-PCR results on nasopharyngeal swabs but did not have a convalescent blood specimen.

[*]MC = Male, castrated; FS = Female, spayed
[†]Clinical signs were reported since onset of illness in the first human case in the household. The duration of clinical signs was unknown.
[+]Blood samples were unable to be obtained for these pets on day 21

Table S2. Owner-reported clinical signs among household pets enrolled in the COVID-19 Household Transmission Investigation since onset of illness in first household human case, April–May 2020.

**Clinical signs**	**Total N=56 n (%)**	**Seropositive N=8 n (%)**	**Seronegative N=39 n (%)**	**No blood sample collected N=9 n (%)**
None	42 (75)	6 (75)	31 (79)	5 (55)
Any signs reported	14 (25)	2 (25)	8 (21)	4 (44)
Respiratory	9 (16)	1 (13)	6 (15)	3 (33)
Sneezing	4 (7)	0 (0)	2 (5)	2 (22)
Coughing	4 (7)	0 (0)	2 (5)	2 (22)
Nasal discharge	3 (5)	1 (13)	2 (5)	0 (0)
Difficulty breathing / shortness of breath	1 (2)	0 (0)	1 (3)	0 (0)
Gastrointestinal	3 (5)	0 (0)	3 (8)	0 (0)
Vomiting	1 (2)	0 (0)	1 (3)	0 (0)
Diarrhea	2 (4)	0 (0)	2 (5)	0 (0)
Other	2 (4)	1 (13)	0 (0)	1 (11)
Inappetence	1 (2)	1 (13)	0 (0)	0 (0)
Lethargy	1 (2)	0 (0)	0 (0)	1 (11)
